# *β*-oxidation–polyhydroxyalkanoates synthesis relationship in *Pseudomonas putid*a KT2440 revisited

**DOI:** 10.1007/s00253-023-12413-7

**Published:** 2023-02-10

**Authors:** Si Liu, Tanja Narancic, Jia-Lynn Tham, Kevin E. O’Connor

**Affiliations:** 1 UCD Earth Institute and School of Biomolecular and Biomedical Science, University College Dublin, Belfield, Dublin 4, Ireland; 2 BiOrbic - Bioeconomy Research Centre, Ireland, University College Dublin, Belfield, Dublin 4, Ireland

**Keywords:** *Pseudomonas putida*, Polyhydroxyalkanoate, *β*-oxidation pathway, Enzyme redundancy

## Abstract

**Abstract:**

*Pseudomonas putida* KT2440 is a well-known model organism for the medium-chain-length (mcl) polyhydroxyalkanoate (PHA) accumulation. (*R*)-Specific enoyl-coenzyme A hydratase (PhaJ) was considered to be the main supplier of monomers for PHA synthesis by converting the *β*-oxidation intermediate, trans-2-enoyl-CoA to (*R*)-3-hydroxyacyl-CoA when fatty acids (FA) are used. Three PhaJ homologues, PhaJ1, PhaJ4 and MaoC, are annotated in *P. putida* KT2440. To investigate the relationship of fatty acids–PHA metabolism and the role of each PhaJ in PHA biosynthesis in *P. putida* KT2440, a series of *P. putida* KT2440 knockouts was obtained. PHA content and monomer composition in wild type (WT) and mutants under different growth conditions were analysed. PhaJ4 was the main monomer supplier for PHA synthesis with FA as sole carbon and energy source, with preference towards C8 and C10 substrate, whereas PhaJ1 showed preference for the C6 substrate. However, when all three PhaJ homologues were deleted, the mutant still accumulated PHA up to 10.7% of the cell dry weight (CDW). The deletion of (*R*)-3-hydroxydecanoyl-ACP:CoA transacylase (PhaG), which connects de novo FA and PHA synthesis pathways, while causing a further 1.8-fold decrease in PHA content, did not abolish PHA accumulation. Further proteome analysis revealed quinoprotein alcohol dehydrogenases PedE and PedH as potential monomer suppliers, but when these were deleted, the PHA level remained at 2.2–14.8% CDW depending on the fatty acid used and whether nitrogen limitation was applied. Therefore, it is likely that some other non-specific dehydrogenases supply monomers for PHA synthesis, demonstrating the redundancy of PHA metabolism.

**Key points:**

• *β-oxidation intermediates are converted to PHA monomers by hydratases PhaJ1, PhaJ4 and MaoC in Pseudomonas putida KT2440.*

• *When these are deleted, the PHA level decreases, but it is not abolished.*

• *PHA non-specific enzyme(s) also contributes to PHA metabolism in KT2440.*

## Introduction

**Fig. 1:**
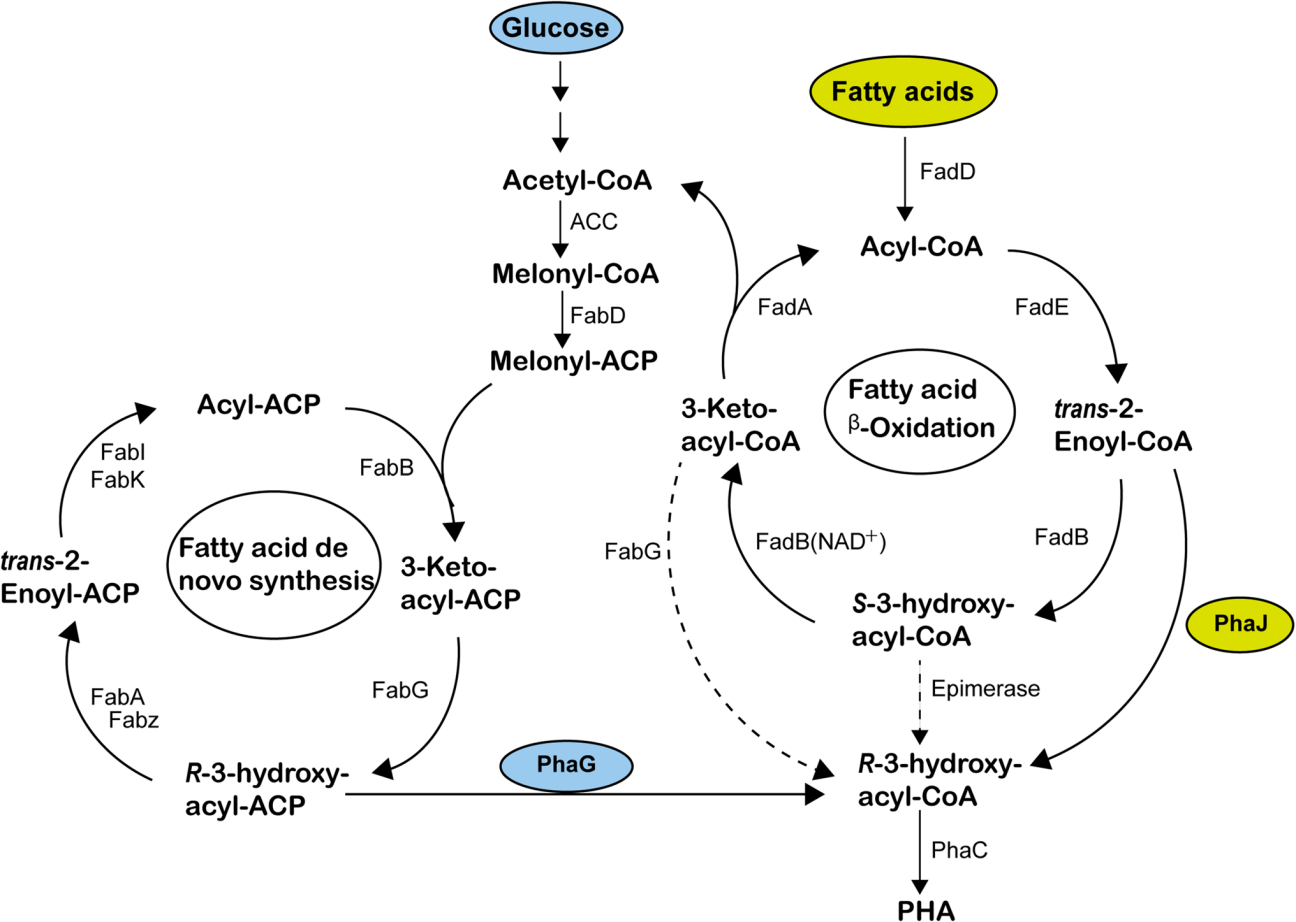
PHA synthesis pathways in bacteria. FadD, acyl-CoA ligase; FadE, acyl-CoA dehydrogenase; FadB, enoyl-CoA hydratase; FadB(NAD^+^), NAD^+^ dependent (*S*)-3-hydroxyacyl-CoA dehydrogenase; FadA, 3-ketoacyl-CoA thiolase; PhaJ, (*R*)-specific enoyl-CoA hydratase; Epimerase, 3-hydroxyacyl-CoA epimerase; ACC, acetyl-CoA carboxylase; FabD, malonyl-CoA:ACP transferase; FabB, 3-ketoacyl-ACP synthase; FabG, 3-ketoacyl-ACP reductase; FabA and FabZ, 3-hydroxyacyl-ACP dehydratase; FabI and FabK, enoyl-ACP reductase; PhaG, 3-hydroxyacyl-ACP:CoA transferase; PhaC, PHA polymerase; PhaZ, PHA depolymerase. Dashed lines indicate uncertain pathways or reactions that are theoretically possible but have not been confirmed

Two main routes for medium-chain-length polyhydroxyalkanoate (mcl PHA) synthesis in *Pseudomonas* strains are known: the *β*-oxidation pathway, when fatty acids are used, and de novo fatty acid synthesis when unrelated substrates such as sugars are used as carbon and energy source (Madison and Huisman 1999) (Fig. 1). (*R*)-Specific enoyl-CoA hydratase PhaJ, which catalyses stereospecific hydration of *trans*-2-enoyl-coenzyme A (enoyl-CoA), an intermediate of *β*-oxidation, was identified as the main supplier of (*R*)-3-hydroxyalkanoyl-CoA monomers for the mcl PHA synthesis from fatty acids (Fiedler et al. 2002; Sato et al. 2011). In *Pseudomonas putida* KT2440 two PhaJ homologues, namely PP_4552 (PhaJ1), and PP_4817 (PhaJ4), were confirmed to be involved in PHA accumulation from fatty acids (Sato et al. 2011). PhaJ1 and PhaJ4 show different preference for mcl PHA precursors: PhaJ4 appears to have a higher preference for 3-hydroxydecanoate (3HD) and 3-hydroxydodecanoate (3HDD) (Sato et al. 2011). Another enzyme, annotated as MaoC (PP_0580), which shows 55% amino acid identity with PhaJ3 homologue from *Pseudomonas aeruginosa*, was hypothesised to contribute to a lower extent to PHA accumulation (Sato et al. 2011). However, it seems that PhaJ4 has the main role in PHA biosynthesis during growth with fatty acids, since only *phaJ4* was expressed when *P. putida* KT2440 was grown with dodecanoic acid (Wang and Nomura 2010).

Two additional channels for the supply of (*R*)-3-hydroxyalkanoyl-CoA monomers were proposed to be 3-ketoacyl-CoA reductase (FabG) acting on 3-ketoacyl-CoA, and an epimerase acting on (*S*)-3-hydroxyalkanoyl-CoA substrate (Fiedler et al. 2002) (Fig. 1). However, it was found that FadBA complex involved in the *β*-oxidation in *Pseudomonads* does not have the epimerase activity and therefore does not provide monomers for PHA (Fiedler et al. 2002). On the other hand, the putative role of FabG in PHA biosynthesis was not clarified. When overexpressed, FabG seems to negatively affect PHA biosynthesis, while PhaJ overexpression yields an increased PHA level (Vo et al. 2008). A possible explanation for this effect is that FabG catalyses a reversible reaction (Vo et al. 2008). It is worth mentioning that FabG has a role in fatty acid biosynthesis where it catalyses reduction of 3-ketoacyl-ACP into (*R*)-3-hydroxyacyl-ACP (Wang et al. 2018).

The 3-hydroxyacyl-CoA-acyl carrier protein transferase PhaG in *P. putida* directly links the fatty acids de novo synthesis and PHA accumulation by converting (*R*)-3-hydroxyacyl-ACP to (*R*)-3-hydroxyacyl-CoA. It is expressed in various *Pseudomonas* species during PHA biosynthesis from an unrelated carbon source (Fiedler et al. 2000; Hoffmann et al. 2002; Hoffmann and Rehm 2004; O'Leary et al. 2005; Rehm et al. 1998; Zheng et al. 2005), and seems to be strongly induced under nitrogen-limited growth conditions (Hoffmann and Rehm 2004). However, the analysis of the transcriptome of KT2440 showed an increase in *phaG* expression when the strain was cultivated with oleic acid, suggesting its potential involvement in PHA accumulation from related substrates (Mozejko-Ciesielska et al. 2018).

While some of the PhaJ homologues were biochemically characterised and the dominant role of PhaJ4 was shown (Sato et al. 2011), it remains unclear what is the role of the remaining PhaJ homologues in *P. putida* KT2440 and their combined effect. To investigate the role of the annotated *phaJ* homologues, we have generated single PhaJ deletion strains, as well as a double *ΔΔphaJ1phaJ4* and a triple knockout *ΔΔΔphaJ1maoCphaJ4* and we have investigated the effect of these deletions on growth, PHA accumulation and PHA monomer composition. We have then analysed the *fabG* expression in the triple knockout background to understand the potential role of this enzyme in supplying PHA monomers. Finally, we have investigated the effect of *phaJ* deletions on the whole proteome of *P. putida* KT2440.

## Materials and methods

### Strains, plasmids and culture conditions

The strains and plasmids used in this study are shown in Table S1. *E. coli* DH5α and *P. putida* KT2440 were grown routinely in Luria Bertani (LB) broth at 37 °C and 30 °C respectively. Carbenicillin (Carb, 50 μg/ml), kanamycin (Km, 50 μg/ml), gentamicin (Gent, 50 μg/ml) and tetracycline (Tet, 10 μg/ml for *E. coli*, 25 μg/ml for *P. putida*) were used as selection antibiotics when needed. All strains were maintained in LB medium with 25% glycerol at − 80 °C.

The minimal salt medium (MSM) (Schlegel et al. 1961) was used for PHA accumulation experiments. A single colony of *P. putida* KT2440 strain from LB plate was inoculated in 3 ml of MSM (non-limiting nitrogen, 1 g/l NH_4_Cl) with 1.95 g of carbon per litre (g_c_/l) of a fatty acid: sodium octanoate (C8), sodium nonanoate (C9), sodium decanoate (C10) or sodium dodecanoate (C12) and incubated for 16 h at 30 °C, with shaking of 200 rpm. The overnight culture was diluted with MSM to get an OD_600nm_ of 1, after which 1 ml of diluted culture was inoculated into 50 ml MSM with ether 1 g/l NH_4_Cl (non-limiting nitrogen) or 0.25 g/l NH_4_Cl (limiting nitrogen) and supplemented with 1.95 gc/l of fatty acid in 250-ml Erlenmeyer flasks. After 48 h of incubation, cells were harvested by centrifugation at 7800 *×g* at 4 °C for 10 min (benchtop 5430R Eppendorf centrifuge, Germany). The cell pellet was washed with 1 ml of di H_2_O and freeze-dried.

### Quantification of PHA and analysis of PHA monomer composition

The PHA content of cells was determined by subjecting approximately 10 mg of lyophilized cells to acidic methanolysis according to a previously described protocol (Lageveen et al. 1988). The resultant 3-hydroxyalkanoic acid (R3HA) methyl esters were assayed by 7890B/5977A Series Gas Chromatograph/Mass Selective Detector (Agilent Technologies, UK) equipped with an HP-5MS column (30 m × 250 μm, 0.25 79-μm-thick film phase, Agilent Technologies, USA) with an oven method of 50 °C for 3 min, increasing by 10 °C/min to 250 °C and holding at this temperature for 1 min. Commercially available R3HA (Sigma-Aldrich, Ireland) were methylated as described above for PHA samples and used as standard to identify the peak. Total PHA content was determined as a percentage of cell dry weight (CDW).

### RNA isolation and cDNA synthesis

Two millilitres of cell culture was harvested from 50 ml of MSM medium in 250-ml flasks at exponential phase. Cell pellets were obtained by centrifugation for 3 min at 12,000 *×g*, 4 °C; the supernatant was completely removed by micropipette. Cell pellets were immediately frozen in liquid nitrogen and maintained at − 80 °C. The total RNA was isolated using GeneJET RNA Purification Kit (Thermal Scientific, USA). DNA contamination in RNA samples was removed by DNases digesting using TURBO DNase (Thermal Scientific, USA). The concentration of pure total RNA was determined with BioDrop μLite (Labplan, Kildare, Ireland). One microgram of total RNA was used for cDNA synthesis with GoScript™ Reverse Transcription System (Promega, USA). cDNA synthesis was performed with preheating of the mixture of random primers, Oligo(dT)15 primer and RNA at 70 °C for 5 min; subsequently, 10 μl of transcription reaction mix was added to the RNA and primer mix for a final reaction volume of 20 μl per tube. The reaction conditions were started with annealing at 25 °C for 5 min, followed by 1-h extension at 42 °C. Finally, the reverse transcriptase was inactivated via incubating reactions at 70 °C for 15 min. The formed cDNA was then diluted by adding 80 μl of DNase/RNase-free water into each reaction and then stored at −80 °C. All procedures using kits were performed according to manufacturers’ instructions. All experiments were performed in triplicates.

### Determination of gene expression level with qPCR

Two reference genes, 16S rRNA processing protein RimM (Gulez et al. 2014; Li et al. 2010) and flagellar protein Flis (Wang et al. 2011; Yu et al. 2018) in *P. putda* KT2440 were selected. The most stable reference gene, Flis, was used for statistical calculation in this study. All primers were designed with primer 3 (http://bioinfo.ut.ee/primer3-0.4.0/primer3/input.htm) and are listed in Table S2. The qPCRs were performed in 12.5-μl reaction mixture containing 1.25 μl of cDNA, 0.2 μM each primer, 6.25 μl TB green DNA polymerase (Takara Bio Europe), and 0.25 μl ROX reference dye II (Takara Bio Europe). The PCR cycling conditions were as follows: initial denaturation at 95 °C for 30 s, followed by 40 cycles of 3-s denaturation at 95 °C and 20 S annealing at 60 °C. Finally, the melt curve was formed during the denaturation at 95 °C for 15 s, followed by cooling of the PCR product at 60 °C for 1 min. In the end of the PCR run, the fluorescence signals were measured continuously as temperature gradually increased from 60 to 95 °C at a speed of 0.05 °C per second. The temperature was held at 95 °C for 15 s. The amplifications were carried out in 74 QuantStudio 7 Flex Real-Time PCR System (Thermo Fisher Scientific, USA). All reactions were performed in triplicates. The expression level of each gene was calculated by the formula:

Gene expression level = 2^Ct (reference) − Ct (target)^

The Ct value is the cycle number when the fluorescence of a PCR product can be detected above the background signal. The gene expression level is presented as the ratio of the mean Ct value of two reference genes to the Ct value of the target.

### Generation of *P. putida* KT2440 mutants

The genes of interest, including *phaJ1*(PP_4552), *phaJ4*(PP_4817), *maoC*(PP_0580), *phaG*(PP_1408), *pedH*(PP_2679), *pedE*(PP_2674) and *hibch*(PP_1412), in *P. putida* KT2440, were scarlessly deleted using modified CRISPR/Cas9 systems and methodology (Cook et al. 2018; Liu et al. 2022). All primers and DNA sequence of the single guide RNA (sgRNA) sequence used for knocking out mutant generation are listed in Table S2. The sgRNAs were designed using the Synthego CRISPR Design Tool (https://design.synthego.com/#/) to target the sequence of a specific *P. putida* KT2440 gene to be deleted. The deletion was performed as previously described (Liu et al. 2022).

### Complementation of *P. putida ΔΔphaJ1phaJ4 and ΔΔΔphaJ1maoCphaJ4*

The genes *phaJ1*, *phaJ4*, and *maoC* were amplified from genomic DNA of *P. putida* KT2440 wild type (WT) using specific primers (Table S2, Primers for complementation). The PCR product was gel purified and assembled with PCR linearised pBT’Tmcs constitutive expressing vector (Koopman et al. 2010) using NEBuilder® HiFi DNA Assembly. Subsequently, the pBT'T plasmid harbouring a constitutive copy of *phaJ1*, *phaJ4* or *maoC* (pBT_*phaJ1*, pBT_*phaJ4* or pBT_*maoC*) electroporated into *∆∆phaJ1phaJ4* or *∆∆∆phaJ1maoCphaJ4* strains for analysis. The *P. putida* KT2440 wild type, *∆∆phaJ1phaJ4* or *∆∆∆phaJ1maoCphaJ4* strain was transformed with empty pBT'T vector and served as control strains. The generated recombinant strains were used for PHA accumulation as described above.

### Proteome analysis

Wild-type *P. putida* KT2440, as well as *ΔΔphaJ1phaJ4*, *ΔΔΔphaJ1maoCphaJ4* and *ΔΔΔΔΔΔphaJ1maoCphaJ4phaGpedHpedE* deletion strains were grown in 50 ml of MSM medium with sodium octanoate under nitrogen-limited conditions as described above. Cell pellets were harvested from 50 ml of culture by centrifugation at 7800 *×g*, at 4 °C for 10 min at exponential growth phase. Supernatant was completely removed by micropipette. The cell pellets were subsequently prepared for proteomic analysis as previously described (Narancic et al. 2016). Trypsin-digested peptides were purified by ZipTip C18 column and analysed by microcapillary high-performance liquid chromatography (LC)–MS/MS as previously described (Narancic et al. 2018).

For the analysis of the proteome of the *ΔΔΔΔΔΔphaJ1maoCphaJ4phaGpedHpedE* deletion strain and comparison with the wild type, the two strains were cultivated as described above. After purification by ZipTip C18 columns, the samples were loaded onto EvoTips and run on a timsTOF Pro mass spectrometer (Bruker Daltonics, Bremen, Germany) coupled to the EvoSep One system (EvoSep BioSystems, Odense, Denmark). The peptides were separated on a reversed-phase C_18_ Endurance column (15 cm × 150 μm ID, C_18_, 1.9 μm) using the preset 30 SPD method. Mobile phases were 0.1% (v/v) formic acid in water (phase A) and 0.1% (v/v) formic acid in acetonitrile (phase B). The peptides were separated by an increasing gradient of mobile phase B for 44 min using a flow rate of 0.5 μl/min.

The timsTOF Pro mass spectrometer was operated in positive ion polarity with trapped ion mobility spectrometry (TIMS) and parallel accumulation serial fragmentation (PASEF) modes enabled. The accumulation and ramp times for the TIMS were both set to 100 ms, with an ion mobility (1/k0) range from 0.6 to 1.6 Vs/cm. Spectra were recorded in the mass range from 100 to 1700 m/z. The precursor (MS) intensity threshold was set to 2500, and the precursor target intensity set to 20,000. Each PASEF cycle consisted of one MS ramp for precursor detection followed by 10 PASEF MS/MS ramps, with a total cycle time of 1.16 s.

The mass spectrometry proteomics data have been deposited to the ProteomeXchange Consortium via the PRIDE (Perez-Riverol et al. 2022) partner repository with the dataset identifiers PXD037932 and PXD037937.

## Results

### The effect of *phaJ* deletions on growth and PHA accumulation

Using the CRISPR/Cas9 system (Cook et al. 2018), we have generated deletions of each of the three PhaJ homologues as well as double and triple PhaJ deletions in *P. putida* KT2440. We have investigated the effect of these deletions on growth and PHA accumulation when a range of fatty acids were used as a carbon and energy source (Table 1).

**Table 1:** Growth and PHA accumulation of *Pseudomonas putida* KT2440 (wild type, WT) and *phaJ* deletion mutants with a range of fatty acids: octanoic acid (C8), nonanoic acid (C9), decanoic acid (C10) and dodecanoic acid (C12). Biomass is represented as cell dry weight (CDW)

Strain		Substrate							
		C8		C9		C10		C12	
		CDW (g/l)	PHA (%CDW)	CDW (g/l)	PHA (%CDW)	CDW (g/l)	PHA (%CDW)	CDW (g/l)	PHA (%CDW)
N-full	WT	1.96 ± 0.01	27.1 ± 2.2	1.73 ± 0.02	24.9 ± 1.5	2.25 ± 0.02	29.4 ± 0.6	2.06 ± 0.01	16.1 ± 3.8
	*ΔphaJ1*	2.00 ± 0.04	25.5 ± 0.5	1.74 ± 0.01	28.1 ± 1.8	2.04 ± 0.04	29.7 ± 0.6	2.17 ± 0.02	20.7 ± 1.4
	*ΔphaJ4*	1.92 ± 0.01	24.8 ± 0.6	1.90 ± 0.03	16.6 ± 0.8	2.26 ± 0.03	20.3 ± 1.1	1.98 ± 0.05	18.3 ± 1.3
	*ΔmaoC*	1.92 ± 0.03	28.7 ± 0.8	1.70 ± 0.03	25.5 ± 1.5	2.22 ± 0.04	29.7 ± 0.4	2.02 ± 0.01	17.6 ± 0.8
	*ΔΔphaJ1phaJ4*	1.62 ± 0.01	7.7 ± 0.5	1.55 ± 0.02	8.2 ± 0.9	1.53 ± 0.08	12.8 ± 2.1	1.69 ± 0.06	9.8 ± 1.4
	*ΔΔΔphaJ1maoC phaJ4*	1.57 ± 0.01	3.4 ± 0.2	1.44 ± 0.03	2.7 ± 0.0	1.63 ± 0.03	4.6 ± 0.5	1.78 ± 0.01	5.2 ± 0.4
	*ΔΔΔΔphaJ1maoCphaJ4phaG*	1.53 ± 0.03	2.4 ± 0.0	1.40 ± 0.02	2.2 ± 0.2	1.67 ± 0.02	3.4 ± 0.1	1.75 ± 0.04	4.1 ± 0.5
N-limitation	WT	1.10 ± 0.02	49.8 ± 1.8	1.02 ± 00.2	47.2 ± 0.4	1.23 ± 0.03	59.5 ± 0.2	1.08 ± 0.03	45.6 ± 9.2
	*ΔphaJ1*	1.24 ± 0.02	55.6 ± 2.7	0.95 ± 0.02	54.6 ± 1.0	0.94 ± 0.02	59.6 ± 4.3	1.25 ± 0.04	49.8 ± 1.4
	*ΔphaJ4*	1.09 ± 0.03	49.4 ± 1.4	1.36 ± 0.14	40.3 ± 0.4	1.25 ± 0.05	41.3 ± 0.4	0.72 ± 0.04	35.3 ± 0.8
	*ΔmaoC*	1.13 ± 0.02	51.5 ± 3.3	1.05 ± 0.05	49.6 ± 0.7	1.23 ± 0.02	58.6 ± 1.4	1.10 ± 0.03	49.1 ± 1.2
	*ΔΔphaJ1phaJ4*	0.67 ± 0.03	18.7 ± 0.7	0.65 ± 0.03	27.0 ± 2.0	0.59 ± 0.06	25.9 ± 2.3	0.56 ± 0.02	20.6 ± 0.3
	*ΔΔΔphaJ1phaJ4maoC*	0.62 ± 0.02	10.7 ± 0.2	0.60 ± 0.02	18.0 ± 0.9	0.65 ± 0.04	10.1 ± 0.3	0.74 ± 0.01	13.0 ± 0.0
	*ΔΔΔΔphaJ1maoCphaJ4phaG*	0.54 ± 0.00	6.9 ± 0.3	0.60 ± 0.02	14.8 ± 0.6	0.61 ± 0.04	6.3 ± 0.4	0.70 ± 0.01	7.3 ± 0.8

All single *phaJ* knockouts, *ΔphaJ4*, *ΔphaJ1* and *ΔmaoC*, respectively, showed similar residual biomass (bacterial biomass excluding PHA) when compared with the wild-type *P. putida* KT2440 (Table 1). A decrease in PHA level up to 1.5-fold was observed with *P. putida ΔphaJ4* cultivated with fatty acids with and without nitrogen limitation, while knocking out *phaJ*1 and *maoC* alone had no effect on PHA accumulation level (Table 1). This suggests that when one of the *phaJ* homologues is knocked out, other enzymes can take over the role with varying efficiency in supplying PHA monomers.

While knocking out two *phaJ* homologues (*ΔΔphaJ4phaJ1*) had a more profound effect on the PHA accumulation level, decreasing PHA accumulation up to 3.3-fold when compared with the WT, PHA was still accumulated under nitrogen-non-limiting and limiting conditions, reaching between 7.7 and 12.8% and 18.7 and 27% cell dry weight (CDW) (Table 1). To understand if *maoC* homologue supplies (*R*)-3-hydroxyalkanoyl-CoA monomers in the *ΔΔphaJ4phaJ1* background, we have generated a triple *phaJ* knockout *ΔΔΔphaJ1maoCphaJ4*. This caused a dramatic decrease in PHA level, yielding a maximum of 5.2% CDW PHA under nitrogen-non-limiting and up to 18% PHA under nitrogen-limiting conditions (Table 1). However, since PHA was still accumulated, this clearly indicated that there are enzymes other than known PhaJ homologues capable of providing PHA monomers from the related carbon source.

### PHA monomer composition in *phaJ* deletion mutants

**Fig. 2:**
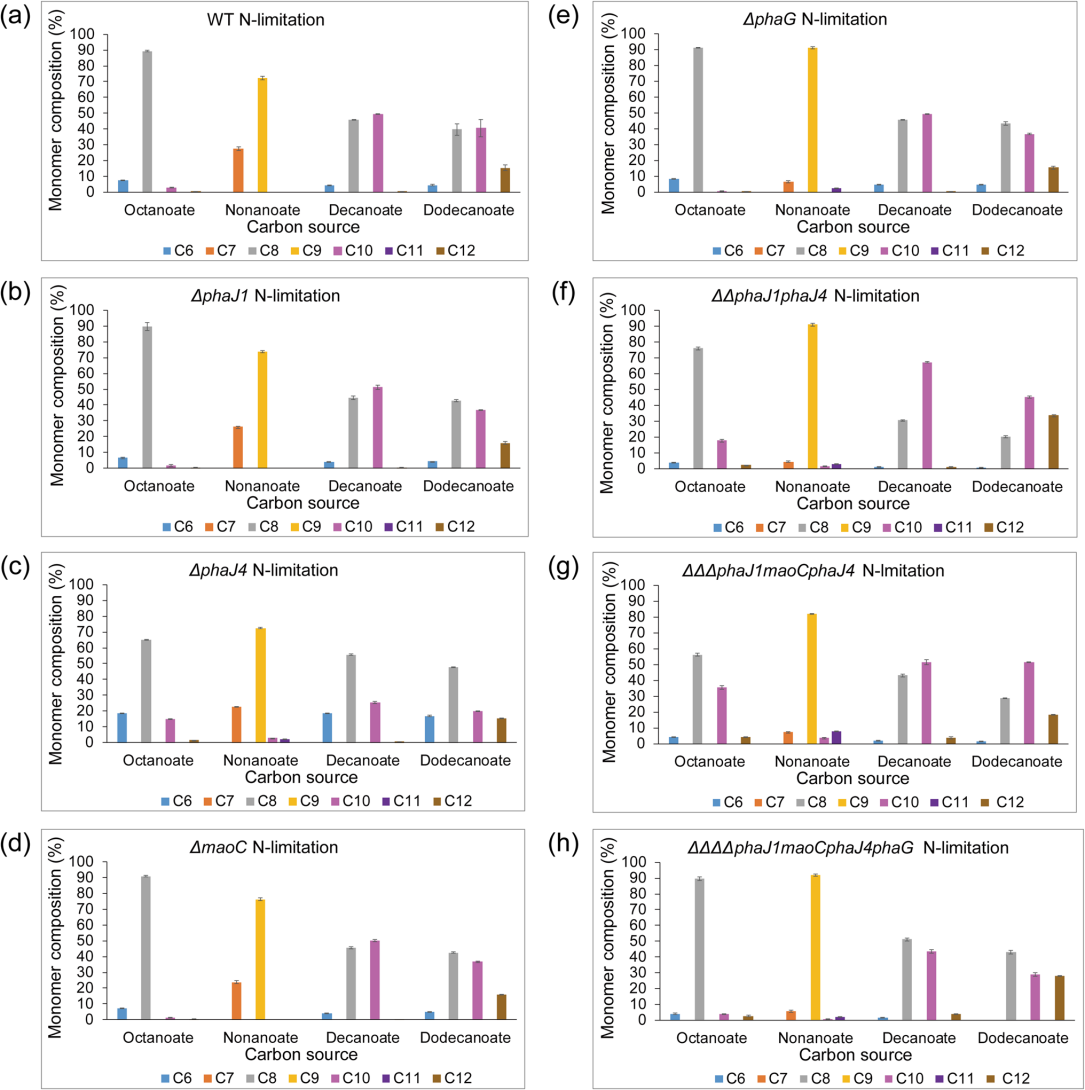
The monomer composition of polyhydroxyalkanoate (PHA) accumulated by *P. putida* KT2440 (**a**), *P. putida ΔphaJ1* (**b**), *P. putida ΔphaJ4* (**c**), *P. putida ΔmaoC* (**d**), *P. putida ΔphaG* (**e**), *P. putida ΔΔphaJ1phaJ4* (**f**), *P. putida ΔΔΔphaJ1maoCphaJ4* (**g**) and *P. putida ΔΔΔΔphaJ1maoCphaJ4phaG* (**h**) when grown with fatty acids under limited nitrogen condition

*ΔphaJ4* mutant always showed an increased C6 (2.4- to 4.2-fold) and C8 (1.2- to 1.4-fold) monomer fraction when compared with the WT (Fig. 2 and Fig. S1). This was accompanied by a 2- to 2.7-fold decrease in C10 fraction when decanoic acid or dodecanoic acids were used as carbon and energy substrates, suggesting that PhaJ4 from *P. putida KT2440* shows preference for C10 substrate. The difference in monomer composition between PHA accumulated by *P. putida ΔphaJ4* and the WT was less profound when nonanoic acid was used as a substrate (Fig. 2 and Fig. S1). When the expression levels of *phaJ1*, *phaJ4*, *maoC*, and *phaG*, the main PHA monomer suppliers, were quantified, *phaJ4* always showed the highest expression level in WT, as well as in the deletion mutants *P. putida ΔphaJ1* and *P. putida ΔmaoC* (Fig. S2 b). The expression was about 3- to 5.9-fold higher than the expression of *phaJ1* and about 15-fold higher than the expression of *maoC* when cells were grown with octanoate under nitrogen limitation (Fig. S2 b). This suggests that in KT2440, the *phaJ4* homologue is the main supplier for PHA synthesis when a PHA-related carbon substrate is used.

The monomer composition of PHA accumulated by *P. putida ΔphaJ1* and *P. putida ΔmaoC* was very similar with the WT PHA (Fig. 2 and Fig. S1). The expression level of *phaJ1* showed no significant change in WT and *ΔmaoC* grown on octanoate (Fig. S2 b). When *phaJ4* was deleted, the expression level of *phaJ1* decreased 1.4-fold compared to the WT level (Fig. S2 b). Nevertheless, *phaJ1* had 2.4-fold higher expression than *maoC* in *ΔphaJ4* mutant (Fig. S2 b). Compared to other tested genes, *maoC* showed the lowest level of expression. However, a 1.3- to 1.4-fold higher expression of *maoC* was observed in the background when *phaJ1* and/or *phaJ4* were deleted (Fig. S2 b), suggesting that *maoC* also can supply monomers for PHA synthesis.

Considering that the C10 monomer fraction was observed regardless of which carbon-related substrate was used, *phaG* expression was also analysed. In the background of *ΔphaJ4* and *ΔmaoC* deletion mutants *phaG* expression increased 2.9-fold compared to the WT strain (Fig. S2 b). However, no significant change in the expression of *phaG* was observed in other strains (WT, *ΔphaJ1*, *ΔmaoC*, *ΔΔphaJ1phaJ4 ΔΔΔphaJ1maoCphaJ4*; Fig. S2 b)*.* This suggests that while the de novo PHA synthesis pathway contributes to PHA synthesis when carbon-related substrates are used, *phaG* does not become a key monomer supplier in the background of multiple *phaJ* deletions mutants, and therefore, other enzymes must be acting as monomer suppliers.

The double-knockout *P. putida ΔΔphaJ1phaJ4* showed decreased C6 and C8 monomer fractions of accumulated PHA when compared with the WT, as well as around 1.4-fold increase in C10 and 2.2- to 3.4-fold increase in C12 fraction when grown with decanoic or dodecanoic acid (Fig. 2).

Finally, the triple knockout showed the same monomer composition as the WT when octanoic or nonanoic acids were used as a substrate, while differences in PHA composition were observed when decanoic or dodecanoic acids were used (Fig. 2). With decanoic acid as a substrate, *P. putida ΔΔΔphaJ1maoCphaJ4* accumulated PHA with 2.5-fold lower C6 and 24-fold higher C12 fractions (Fig. 2). When dodecanoic acid was used as carbon and energy substrate, the highest fraction was C10 (51 mol%), while the WT PHA had equal amounts of C8 and C10 (around 40 mol%, Fig. 2). The differences in monomer composition of the PHA accumulated by the double and triple knockout most likely reflect the substrate preference of an additional enzyme supplying the monomers when *phaJ* homologues are deleted.

Fatty acids, as carbon-related substrates for PHA synthesis, are broken down via *b*-oxidation by cleaving two carbons each cycle. So, only the monomers containing the same or − 2C number carbons with fatty acids feed are expected to be present in PHA, when the beta-oxidation cycle is the sole supply of monomer. However, C10, C11 or C12 monomer fractions were detected by GC–MS when the strains were grown on octanoic, nonanoic or decanoic acid (Fig. 2). This suggests the presence of other pathways that can elongate fatty acids. Compared with the WT, *P. putida ΔphaJ4*, *P. putida ΔΔphaJ1phaJ4* and *P. putidaΔΔΔphaJ1maoCphaJ4* mutant showed 5- to 13-fold increase in C10 fraction and up to 12-fold increase in C12 fraction when grown on octanoic acid, as well as 1.8- to 24-fold increase in C12 when grown on decanoic acid (Fig. 2). We hypothesised that some of the acetyl-CoA generated by beta-oxidation will be shunted towards de novo synthesis and finally converted to *R*-3-hydroxyacyl-CoA by PhaG, and that this effect might be emphasised in *phaJ* deletion strains (Fig. 1). To support this hypothesis, a single-deletion mutant *P. putida ΔphaG* and quadruple mutant *P. putida ΔΔΔΔphaJ1maoCphaJ4phaG* were created. *ΔphaG alone* showed the same growth, PHA accumulation and monomer composition as WT when grown on fatty acids (Table 2, Fig. 2 and Fig. S1), suggesting that PhaG does not play a significant role in PHA accumulation when carbon-related substrates are used and the beta-oxidation pathway is available. However, when PhaJ hydratases were not available to provide the precursors for PHA polymerisation, PhaG seems to play a more prominent role as evidenced from the 1.6–1.8-fold decrease in PHA content when *P. putida ΔΔΔΔphaJ1maoCphaJ4phaG* was grown on fatty acids (Table 1). This conclusion is also supported by the decrease in C10 fraction in *ΔΔΔΔphaJ1maoCphaJ4phaG* compared with *P. putida ΔΔΔΔphaJ1maoCphaJ4* when grown on octanoic, decanoic or dodecanoic acid (Fig. 2).

**Table 2:** Growth and PHA accumulation of *Pseudomonas putida* KT2440 (wild type, WT) and *phaG* deletion mutant with a range of fatty acids: octanoic acid (C8), nonanoic acid (C9), decanoic acid (C10) and dodecanoic acid (C12). Biomass is represented as cell dry weight (CDW)

	Strain	Substrate							
		C8		C9		C10		C12	
		CDW (g/l)	PHA (%CDW)	CDW (g/l)	PHA (%CDW)	CDW (g/l)	PHA (%CDW)	CDW (g/l)	PHA (%CDW)
N-full	WT	1.92 ± 0.04	28.5 ± 1.1	1.75 ± 0.03	33.4 ± 1.7	2.20 ± 0.07	36.7 ± 1.3	2.38 ± 0.03	37.9 ± 1.6
	*ΔphaG*	1.88 ± 0.03	29.1 ± 3.0	1.74 ± 0.02	31.8 ± 1.1	2.10 ± 0.04	34.7 ± 0.9	2.37 ± 0.02	37.3 ± 0.8
N-limitation	WT	1.08 ± 0.02	56.7 ± 2.4	0.88 ± 0.05	56.3 ± 1.0	1.12 ± 0.03	66.2 ± 1.8	1.29 ± 0.08	65.0 ± 1.2
	*ΔphaG*	1.07 ± 0.02	54.5 ± 0.4	0.82 ± 0.00	56.4 ± 2.9	1.05 ± 0.05	66.3 ± 2.1	1.09 ± 0.01	66.9 ± 3.2

### FabG expression in PhaJ deletion mutants

**Fig. 3:**
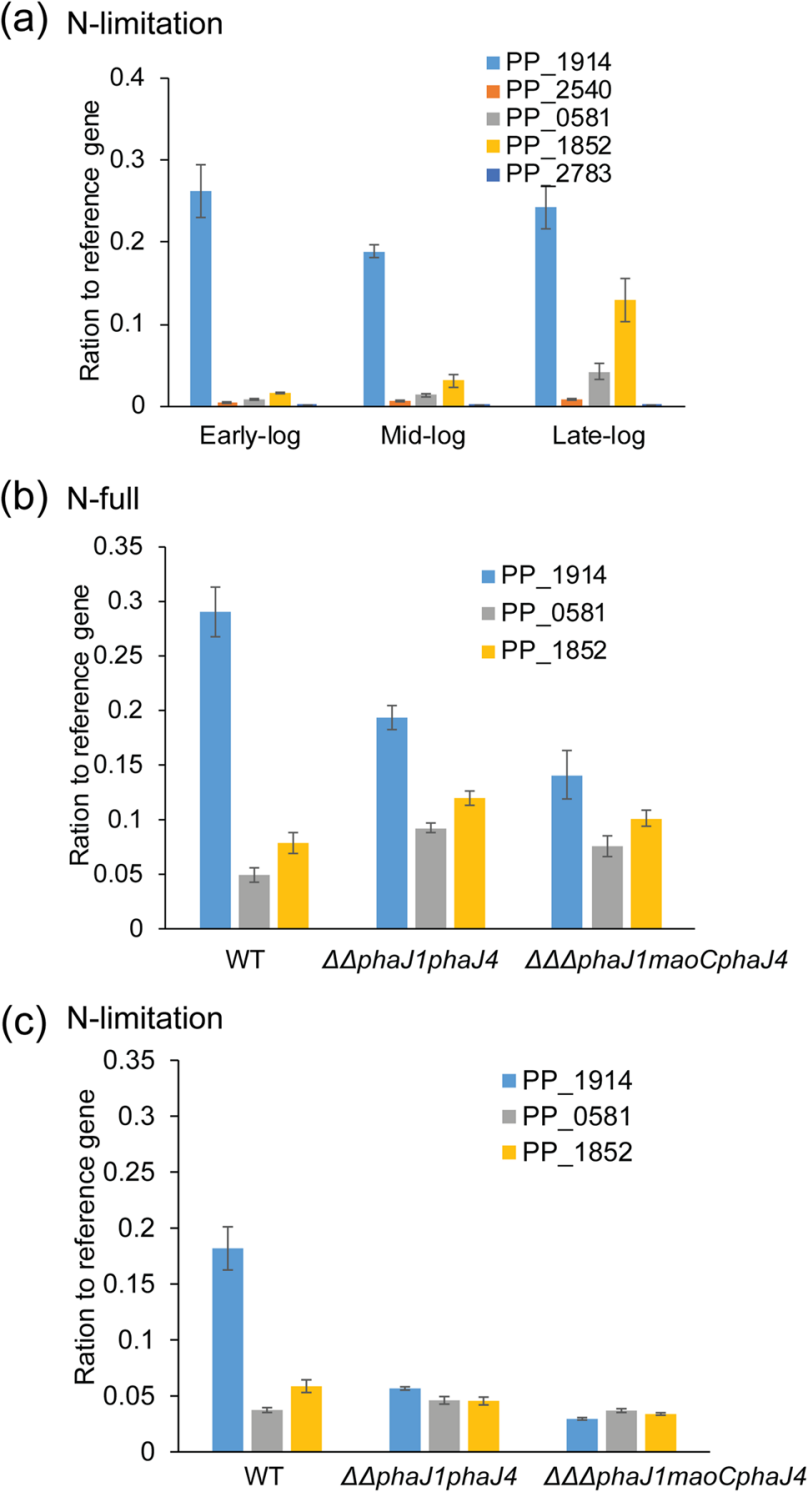
The expression level of *fabG* homologues in *P. putida* when grown in MSM medium with sodium octanoate. **a** Expression level of five isomers of *fabG* in wild-type *P. putida* at exponential phase under limited nitrogen condition. **b** and **c** Comparison of the expression of three *fabG* (PP_1914, PP_0581 and PP_1852) in WT *P. putida*, *P. putida ΔΔphaJ1phaJ4* and *P. putida ΔΔΔphaJ1maoCphaJ4* under N-full (**b**) and N-limitation (**c**) conditions

To understand the potential role of *fabG* in PHA synthesis when *phaJ* homologues are deleted, the expression level of *fabG* homologues in WT or *PhaJ* deletion mutants was determined by qPCR. Five FabG homologues in *P. putida* KT2440 genome based on KEGG PATHWAY database were selected, and they showed 26–39% amino acid similarity (Table S3). Their expression level in WT under non-limited nitrogen condition was firstly analysed (Fig. 3a). PP_1914 showed the highest expression level during the log phase of growth compared with other four *fabG* homologous, while PP_2540 and PP_2783 had very low expression. In addition, PP_0581 showed 4.8-fold increase and PP_1852 had 8.3-fold increase in expression level at late-log phase compared with the early-log phase, respectively. The results indicated that PP_1914, PP_0581 and PP_1852 could potentially contribute to PHA synthesis. Therefore, the gene expression level of these three *fabG* homologous was further analysed in WT, *P. putida ΔΔphaJ1phaJ4* and *P. putida ΔΔΔphaJ1maoCphaJ4* under nitrogen-limiting or non-limiting conditions (Fig. 3b, c). PP_1914 showed 1.2- to 5.5-fold decrease in expression level in both mutants when compared with WT under both nitrogen-limited and non-limited conditions (Fig. 3b, c). Similarly, neither PP_0581 nor PP_1852 showed significant difference in the expression level in the double-deletion mutant and triple-deletion mutant, suggesting that none of the analysed *fabG* homologues are responsible for PHA accumulation in the *phaJ* deletion background (Fig. 3b, c).

### Proteomic investigation of *P. putida* KT2440 wild type, double and triple *phaJ* mutants

Considering that *ΔΔΔphaJ1maoCphaJ4* still accumulates 2.7–5.2% CDW PHA under nitrogen non-limiting, and 10.1–18.0% CDW PHA under nitrogen-limiting conditions (Table 1), and *phaG* does not seem to take over the role of the key monomer supplier, we sought to identify other potential PHA monomer suppliers by analysing the proteome of the *ΔΔΔphaJ1maoCphaJ4*. The proteomes of the WT, *ΔΔphaJ1phaJ4* and *ΔΔΔphaJ1maoCphaJ4* strains were compared, and all proteins with a statistically significant fold change ≥ 2 were further analysed (Table S4).

Two quinoprotein ethanol dehydrogenases, PedH (PP_2679) and PedE (PP_2674), which have been shown to participate in the metabolism of different substrates such as ethylene glycol and n-butanol (Muckschel et al. 2012; Simon et al. 2015; Wehrmann et al. 2017), are highly upregulated in *∆∆phaJ1phaJ4* (226-fold for PedH and 69-fold for PedE) and *∆∆∆phaJ1maoCphaJ4* (89-fold for PedH and 70-fold for PedE) compared to the WT (Table S4). Considering PedH and PedE proteins are redox enzymes, catalysing the conversion between ketone and alcohol and exhibiting a broad substrate range (Wehrmann et al. 2020), we hypothesised these two enzymes could convert 3-ketoacyl-CoA to the monomer (*R*)-3-hydroxyacyl-CoA. To verify this hypothesis, the *pedH* and/or *pedE* gene(s) were deleted in quadruple mutant (*∆∆∆∆phaJ1maoCphaJ4phaG*) to create *∆∆∆∆∆phaJ1maoCphaJ4phaGpedH* and *∆∆∆∆∆∆phaJ1maoCphaJ4phaGpedHpedE*. Then, the PHA accumulation and cell growth of the quadruple, quintuple, and sextuple mutants in MSM medium supplemented with octanoate under both nitrogen-limiting and non-limiting conditions were compared. However, no further decrease in PHA accumulation was observed after *pedH* and *pedE* were deleted (Table 3), meaning PedH and PedE do not contribute to PHA synthesis in *P. putida* KT2440 under these conditions. The reason for the highly elevated expression of these quinoproteins in the background of deleted *phaJ* homologues remains to be determined. The complete list of protein expression profiles in *P. putida* KT2440 wild type, *∆∆phaJ1phaJ4* and *∆∆∆phaJ1maoCphaJ4* when PHA accumulation by addition of sodium octanoate under limited nitrogen condition can be found in supplementary information (Table S7).

**Table 3:** Growth and PHA accumulation of *ΔΔΔΔphaJ1maoCphaJ4phaG*. *ΔΔΔΔΔphaJ1maoCphaJ4phaGpedH*, *ΔΔΔΔΔΔphaJ1maoCphaJ4phaGpedHpedE* and *ΔΔΔΔΔphaJ1maoCphaJ4phaGhibch* mutants. Cells were cultivated for 48 h at 30 °C in MSM medium supplemented with 3.37 g/l sodium octanoate under full nitrogen (N-full) and nitrogen limitation (N-limitation) conditions. Biomass is represented as cell dry weight (CDW). All experiments were carried out in triplicate. C6, (*R*)-3-hydroxyhexanoate; C8, (*R*)-3-hydroxyoctanoate; C10, (*R*)-3-hydroxydecanoate; C12, (*R*)-3-hydroxydodecanoate

Medium	Strains	CDW (g/l)	PHA content (% CDW)	PHA composition (mol%) C6:C8:C10:C12
N-full	*ΔΔΔΔphaJ1maoCphaJ4phaG*	1.44 ± 0.06	3.5 ± 0.58	9:76:15:0
	*ΔΔΔΔΔphaJ1maoCphaJ4phaGpedH*	1.41 ± 0.08	4.08 ± 0.03	8:76:15:0
	*ΔΔΔΔΔΔphaJ1maoCphaJ4phaGpedHpedE*	1.37 ± 0.10	4.52 ± 0.03	7:80:13:0
	*ΔΔΔΔΔphaJ1maoCphaJ4phaGhibch*	1.40 ± 0.05	4.06 ± 1.01	8:76:15:0
N-limitation	*ΔΔΔΔphaJ1maoCphaJ4phaG*	0.50 ± 0.00	8.15 ± 0.18	6:88:6:0
	*ΔΔΔΔΔphaJ1maoCphaJ4phaGpedH*	0.50 ± 0.01	8.34 ± 0.57	6:88:6:0
	*ΔΔΔΔΔΔphaJ1maoCphaJ4phaGpedHpedE*	0.54 ± 0.05	7.54 ± 2.07	6:87:7:0
	*ΔΔΔΔΔphaJ1maoCphaJ4phaGhibch*	0.47 ± 0.01	8.83 ± 0.55	6:88:6:0

Finally, we have analysed the proteome of the sextuple mutant under nitrogen-limiting (Table S5) and non-limiting conditions (Table S6) and identified several non-specific hydratases that potentially could act as suppliers for PHA monomers. Based on the STRING interaction network analysis, and using KT2440 PhaJ homologues for the search, other hydratases, such as PP_3726, PP_1845, PP_2217, PP_2136 and PP_3284, PP_3491, PP_3925 were identified. We have detected PP_1845, PP_2217, PP_2136, PP_3925 and PP_3491 in the proteome of both the WT and sextuple mutant, albeit without a statistically significant change in the expression level among the tested conditions (octanoate, with or without nitrogen limitation; Supplemental information). However, there is a trend of increase in PP_2217 expression in the sextuple mutant compared to the WT, but due to the variation among biological replicates, this was not found to be a statistically significant change.

## Discussion

It this study, the *R*-specific enoyl-CoA hydratase route was demonstrated as the main *R*-3-hydroxyacyl-coA supplier (at least 62% contribution) for PHA synthesis in *Pseudomonas putida* KT2440 when fatty acids are used as sole carbon and energy source (Table 1). Both *phaJ1* and *phaJ4* are *R*-specific enoyl-CoA hydratases (Table 1); however, *phaJ4* is the key contributor of monomers in *P. putida* KT2440 (Fig. S3). The key role of PhaJ4 was also shown in *E. coli* harbouring *phaJ1* or *phaJ4* with *phaC*, where 19% and 31% of PHA was accumulated, respectively, when grown on sodium dodecanoate (Sato et al. 2011). The enoyl-CoA hydratase encoded by *phaJ4* in *P. putida* strain A514 grown on vanillic acid is stress-induced and likely to contribute to PHA synthesis under nitrogen-starvation conditions (Wang et al. 2018). Our study also demonstrated that the protein encoded by *maoC* (PP0580) in KT2440, showing 55% identity (of 251 aa) to *phaJ3* from *Pseudomonas aeruginosa* (Sato et al. 2011), contributes to PHA accumulation in *P. putida* KT440, albeit at a lower extent compared to *phaJ1* and *phaJ4* (Table 1 and Fig. S3). The overexpression of single *phaJ* homologues in KT2440 had no effect on PHA level or monomer composition when the recombinant strains were grown with octanoate, and no limitation was applied (Table S8). However, under nitrogen limitation, the PHA level decreased up to 1.9-fold compared to the control (KT2440 carrying an empty vector), with no effect on monomer composition (Table S8). This finding is contrary to the previously reported increase in PHA level upon *phaJ* overexpression in a *P. putida* KCTC1639 (Vo et al. 2008), and suggests that simply an overexpression of the key supplier of PHA monomers, without the accompanying increase of the substrate, has a negative effect on PHA accumulation, and further confirms the role of PHA metabolism as an efficient regulator of carbon and energy balance in KT2440. Another possible explanation is that PhaJ homologues when present at high levels may exhibit a reverse reaction, thereby removing the PHA monomer available for the polymerisation.

KT2440 was still able to accumulate a substantial amount of PHA when all three PhaJ hydratases, i.e. PhaJ1, PhaJ4 and MaoC/PhaJ3, were deleted. Since the role of FabG in PHA accumulation in KT2440 was not clarified, and its capacity to reduce mcl-3-ketoacyl-CoAs to 3-hydroxyacyl-CoAs in *E. coli* and *P. aeruginosa* was demonstrated (Nomura et al. 2008; Ren et al. 2000; Taguchi et al. 1999), we decided to revisit FabG expression in *phaJ* deletion mutants. However, neither of the tested *fabG* homologues showed increased expression in *P. putida ΔΔphaJ1phaJ4* and *P. putida ΔΔΔphaJ1maoCphaJ4* compared to *P. putida* WT (Fig. 3). Furthermore, overexpression of FabG (99% homology with PP_1914) in *P. putida* KCTC1693 leads to the decrease of PHA content due to reversible conversion of (*R*)-3-hydroxyalkanoate monomer units into 3-ketoacyl-CoA (Vo et al. 2008). To definitely rule out the role of FabG homologues in PHA synthesis in KT2440, deletion of these genes was attempted; however, we were not able to obtain viable mutants. This was similarly observed when the PP_1914 homologue was deleted in *E. coli* or *P. aeruginosa* causing cell death (Ren et al. 2000; Zhang and Cronan 1998).

While it is known that PhaG expression is increased under a nitrogen-limiting condition, and that this is the main monomer supplier when carbon-unrelated substrates are used (Fig. S2) (Hoffmann and Rehm 2004; Steinbüchel 2001), it was suggested that PhaG also has a role in PHA accumulation when carbon-related substrates are used. PhaG indeed showed elevated expression in *phaJ4* and *maoC* single-deletion mutants compared to the WT and other mutants under nitrogen-starvation condition when grown on octanoate (Fig. S2). But, when we deleted *phaG* in the background of *phaJ* mutants, there was still 6.3–14.8% of PHA accumulated when fatty acids were used (Table 1). While PhaG contributes to a lower extent to PHA accumulation when related substrates are used, the *ΔphaG* mutant showed no significant difference in biomass, PHA level or monomer composition when fatty acids were used (Table 2; Fig. 2a, e), ruling out this route to take over the monomer supply. A PhaG homologue, PP_0763, annotated as a mcl-fatty acid ligase, when heterologously expressed in *E. coli* in addition to *phaC*, leads to PHA accumulation (Wang et al. 2012). We indeed found PP_0763 to be expressed in the WT and in the sextuple mutant; however, there was no significant difference in the expression level among the strains and cultivation conditions (Supplemental information).

It then appeared that quinoprotein dehydrogenases encoded by *pedH* (PP_2979) and *pedE* (PP_2674) could have a role in PHA accumulation when other known monomer supplying routes were intercepted, based on the significantly elevated expression of PedH and PedE in the double- and triple-deletion mutants (Table S4). These periplasmic enzymes have been associated with oxidation of a range of alcohols and aldehydes (Thompson et al. 2021; Wehrmann et al. 2017; Wehrmann et al. 2020). Considering their broad selectivity, we hypothesised that PedE and/or PedH may be involved in PHA metabolism, i.e. non-specifically reduce 3-ketoacyl-CoA into (*R*)-3-hydroxyacyl-CoA. However, a sextuple mutant *ΔΔΔΔΔΔphaJ1maoCphaJ4phaGpedHpedE* was still able to accumulate PHA when octanoate was used as a carbon and energy substrate. It was suggested that PedE and PedH could complement each other’s activity if PedE, a cytochrome *c* involved in PQQ regeneration, was intact (Thompson et al. 2021). The sextuple mutant contains both of these dehydrogenases deleted, and therefore proves that these enzymes are not involved in the PHA metabolism. The reason for such elevated expression of PedE and PedH when main PHA monomer suppliers were deleted remains unclear.

While it was hypothesised that epimerase activity of FadB could also be a route to (*R*)-3-hydroxyacyl-CoA, it appears that, at least in *Pseudomonas* strains, this is not the case (Fiedler et al. 2002). Many studies have shown that the enzymes of *β*-oxidation are redundant. Several homologues of FadBA and FadD, as well as acyl-CoA dehydrogenases, have been described (Mezzina et al. 2021). It is possible that the same is true for PHA synthesis, and that there is “PHA synthesis redundancy”. Our study suggests it is likely that some other dehydrogenases or hydratases could non-specifically reduce 3-ketoacyl-CoA, or hydrate 2-*trans*-enoyl-CoA intermediate and provide monomers for PHA polymerisation. The monomer composition of PHA accumulated by the sextuple mutant is high, 80–87 mol% of the C8 monomer when octanoate was used as a sole carbon and energy source, which is very similar to the WT monomer composition (Table 3; Fig. 2). For example, in *P. aeruginosa*, in addition to four PhaJ homologues, an enoyl-CoA hydratase complex RhlYZ involved in rhamnolipid biosurfactants biosynthesis was found to also catalyse the conversion of trans-2-decenoyl-CoA to (*R*)-3-hydroxydecanoyl-CoA (Abdel-Mawgoud et al. 2014). This enoyl-CoA hydratase RhlYZ showed 67.4% of identity with the 3-hydroxisobutyryl-CoA hydrolase (HIBCH) encoded by PP_1412 in KT2440 strain. However, we have not detected this protein in any of the tested strains under any of the tested conditions (Supplemental information). When this gene was deleted in the background of the triple *ΔΔΔphaJ1maoCphaJ4* mutant, no change in PHA level or monomer composition was observed (Table 3), suggesting that this enzyme plays no role as an additional PHA monomer supplier.

The redundancy of enzymes involved in PHA metabolism formulates the question of the importance of PHA metabolism. While the physiological role of PHA is not vital, it was shown that removing the capacity to accumulate PHA by deleting the PhaC1 polymerase caused morphological changes, i.e. reduced size of cells (De Eugenio et al. 2010a; De Eugenio et al. 2010b). This phenomenon was explained by a regulatory role of PHA metabolism in maintaining the carbon and energy balance (Escapa et al. 2012; Manoli et al. 2022). KT2440 natively produces large amounts of reduced equivalents (Blank et al. 2008), while PHA acts as a sink for these reduced equivalents. Furthermore, the dynamic nature of PHA metabolism and simultaneous polymerisation and depolymerisation seem to ensure the optimal carbon capture, as the removal of PHA synthesis leads to carbon spillage via increased respiration rate (Escapa et al. 2012). This “buffering” role of PHA metabolism has been recently exploited to control the carbon flow in KT2440 (Manoli et al. 2022). Therefore, considering this important physiological role of PHA in balancing the energy status of KT2440, the redundancy in PHA monomer supplying function is a likely scenario.

## Data Availability

All data and strains are available upon request.
